# Potential roles of gangliosides in chemical-induced neurodegenerative diseases and mental health disorders

**DOI:** 10.3389/fnins.2024.1391413

**Published:** 2024-04-22

**Authors:** Yutaka Itokazu, Alvin V. Terry

**Affiliations:** ^1^Department of Pharmacology and Toxicology, Medical College of Georgia at Augusta University, Augusta, GA, United States; ^2^Department of Neuroscience and Regenerative Medicine, Medical College of Georgia at Augusta University, Augusta, GA, United States

**Keywords:** chemical warfare, cholinergic system, ganglioside, mental health disorder, neurodegenerative disease, neurotoxicity, organophosphate, pesticide

## 1 Introduction

The prevalence of neurodegenerative diseases and mental disorders have been increasing over the past few decades. While genetic and lifestyle factors are important to the etiology of these illnesses, the pathogenic role of environmental factors, especially toxicants such as pesticides encountered over the life span is receiving increased attention. In addition, some toxicants have been utilized as chemical warfare agents and they may pose a greater risk to human health than biological weapons because of easier access. Chemical warfare agents have been used in World War I and II, the Iran-Iraq War, and other more recent conflicts in the Middle East. Toxic chemicals have also increasingly attracted the attention of terrorists, since these weapons have potentially devastating effects on the general population. As an example, the Japanese terrorist cult Aum Shinrikyo released the nerve agent sarin in the Tokyo subway in 1995 killing 13 people and causing more than 5,800 people to seek medical attention. In addition, there have been a number of accidental toxicant exposures during storage and shipments of hazardous chemicals over the years including a recent example, the Ohio train derailment in 2023. It is thus important to establish more effective medical countermeasures to improve recovery from toxicant exposures to counter an ever present environmental health concern (National Toxicology Program, 2019; Reddy, 2024).

Among the toxicants found in many pesticides and chemical warfare agents, the chemicals known as organophosphates are most frequently associated with adverse long-term neurological consequences (Figueiredo et al., 2018; Naughton and Terry, 2018). Organophosphates are defined as any organic compound whose molecule contains phosphate ester groups. In addition to pesticides and chemical warfare agent organophosphorus compounds have been utilized as fuel additives, plasticizers, flame retardants, lubricants, and pharmaceuticals. Their primary mechanism of acute neurotoxicity is based on the irreversible inhibition of acetylcholinesterase which is the major enzyme responsible for hydrolysis of acetylcholine in the cholinergic synapse. In the body, acetylcholinesterase prevents overstimulation of postsynaptic neurons by reducing levels of acetylcholine available to the cholinergic receptor. However, as a result of organophosphorus exposure, the breakdown of acetylcholine is prevented, resulting in a state of sustained excitation of cholinergic receptors. Organophosphate chemical agents cause severe convulsions and seizures, leading to severe damage of the central nervous system (CNS). Antidotal therapy is available to combat the symptoms of acute organophosphate toxicity, however, there are no established interventions for the long-term neurological consequences of organophosphate exposure. It is thus highly desirable to better understand the mechanisms by which these chemical agents affect the nervous system chronically and lead to dysfunctional neuronal activity.

In the nervous system, lipids are the most abundant organic compounds, and a variety of lipids control the biophysical nature of lipid membranes. Glycosphingolipids are a class of lipids that are unique amphipathic molecules with a hydrophilic carbohydrate portion and hydrophobic lipid component. Gangliosides are sialic acid-containing glycosphingolipids known to play essential roles in cell-cell recognition, adhesion, signal transduction, and cellular migration, and that are crucial in all phases of neurogenesis (Itokazu and Yu, 2023; Itokazu et al., 2023). Mice lacking major glycolipids show lethal phenotypes, indicating the significance of glycolipids to mammalian physiology. Moreover, patients with ganglioside deficiencies exhibit severe clinical phenotypes and altered glycosphingolipid expression has been associated with neurodegenerative diseases and mental health disorders. It is also important to note that alterations in glycolipid composition after organophosphate exposure in mammals and birds have been reported (Rozengart and Taranova, 1969; Taranova, 1978; Islam et al., 1983; Khan and Hasan, 1988; Bush et al., 1995). Here we focus on the potential of glycolipids to protect and promote regeneration of the CNS in chemical-induced neurodegenerative diseases and mental health disorders as a therapeutic strategy.

## 2 Potential functions of gangliosides in chemical-induced neurodegenerative diseases and mental health disorders

### 2.1 Toxic chemicals are associated with neurological and psychiatric disorders

Multiple reports have made associations between environmental organophosphate exposure and neurodevelopmental deficits including impaired cognitive and neurobehavioral development, decreased intelligence quotient (IQ), and increased abnormal reflexes (reviewed, Jokanovic et al., 2023). Both prenatal and postnatal exposure to organophosphates have been associated with cognitive and attentional deficits as well as, attention deficit hyperactivity disorder (ADHD). Occupational exposure to organophosphates has also been associated with elevated levels of anxiety and depression in adults, although children were more sensitive to neurotoxicity caused by organophosphates during brain development. Organophosphorus ester-induced chronic neurotoxicity (OPICN) also called chronic organophosphate-induced neuropsychiatric disorder (COPIND) appears as a set of long-term, persistent, chronic neuropsychiatric symptoms including apathy, decreased visual memory, impaired vigilance, reduced abstract reasoning, anxiety, depression, increased social isolation, reduced fine motor coordination, confusion, dizziness, insomnia, reduced vibrotactile sensitivity, decreased academic skills, emotional lability, irritability, short-term memory deficits, decreased verbal attention, fatigue, problems with concentration, and slowing of reaction time. After single dose exposure to organophosphates in Japan in the Aum Shinrikyo terrorist attack, assault victims had eye-related symptoms, physical fatigue, numb muscles, headache, depressive mood, forgetfulness, and chronic lack of concentration for 5–10 years afterwards (Kawana et al., 2001; Yanagisawa et al., 2006). Despite considerable research to investigate the association between these toxic chemicals and persistent neurological and psychiatric disorders, neuropathologic mechanisms of the long-term impairments by organophosphate exposure remain unclear.

### 2.2 The cholinergic system and neurological and psychiatric diseases

Acetylcholine is the neurotransmitter of the cholinergic system and it has an important role in memory, attention, motivation, novelty seeking and arousal. Dysfunction of the cholinergic system is clinically significant in several diseases and thought to play a major role, in Alzheimer's disease (Terry and Buccafusco, 2003). Acetylcholine acts on two main types of receptors, nicotinic and muscarinic acetylcholine receptors. Neuronal nicotinic acetylcholine receptors (nAChRs) are expressed both pre- and post-synaptically in most regions of the brain (Terry et al., 2023) and it has been reported that dysfunctional muscarinic and nicotinic receptors contribute to the symptoms of multiple neurologic and psychiatric illnesses in addition to AD and include, Parkinson's disease (PD), epilepsy, schizophrenia, and depression. In the brains of patients with AD, nAChR mRNAs are decreased in brain regions that are important for memory and cognition and significant correlations between the levels of the loss of nAChRs in the brain and the degree of cognitive decline in AD have been reported. In the brains of patients with schizophrenia, decreases in the expression of nAChRs have also been observed. Imaging studies have also revealed that nAChR availability in depressed patients across all brain regions that were evaluated was lower than that in healthy controls (Terry et al., 2023).

Gangliosides have been reported to interact with neurotrophin receptors and support neuroprotective phenomena as well as other many essential functions (Fantini and Barrantes, 2009; Ledeen and Wu, 2015; Furukawa et al., 2019) that are important to cholinergic neurons. For example, cholinergic innervations of the rat cortex were decorticated and intracerebroventricular infusions of GM1 showed successful attenuation of reduced choline acetyltransferase levels. This GM1 effect was similar to that of NGF infusion, while a combination infusion of NGF and GM1 had a synergistic effect to significantly increase cholinergic presynaptic terminal size (Garofalo et al., 1993). In non-human primates, administration of NGF alone or in combination with GM1 induced a long-term protective effect on nucleus basalis cholinergic neurons after neocortical infarction. Although a protection of the cholinergic cell bodies was found with both treatments, a significant recovery of the neuritic processes was observed only in the double-treated monkeys (Liberini et al., 1993). A potential mechanism for the positive effects of GM1 described above may be related to its binding to the high affinity NGF receptor, TrkA (Mutoh et al., 1995, 1998). The studies described here indicate that modulation of important components of the molecular environments of cholinergic neurons (via gangliosides) may have broad therapeutic potential.

### 2.3 Altered ganglioside composition in chemical agent exposure and in CNS diseases

**Figure 1:**
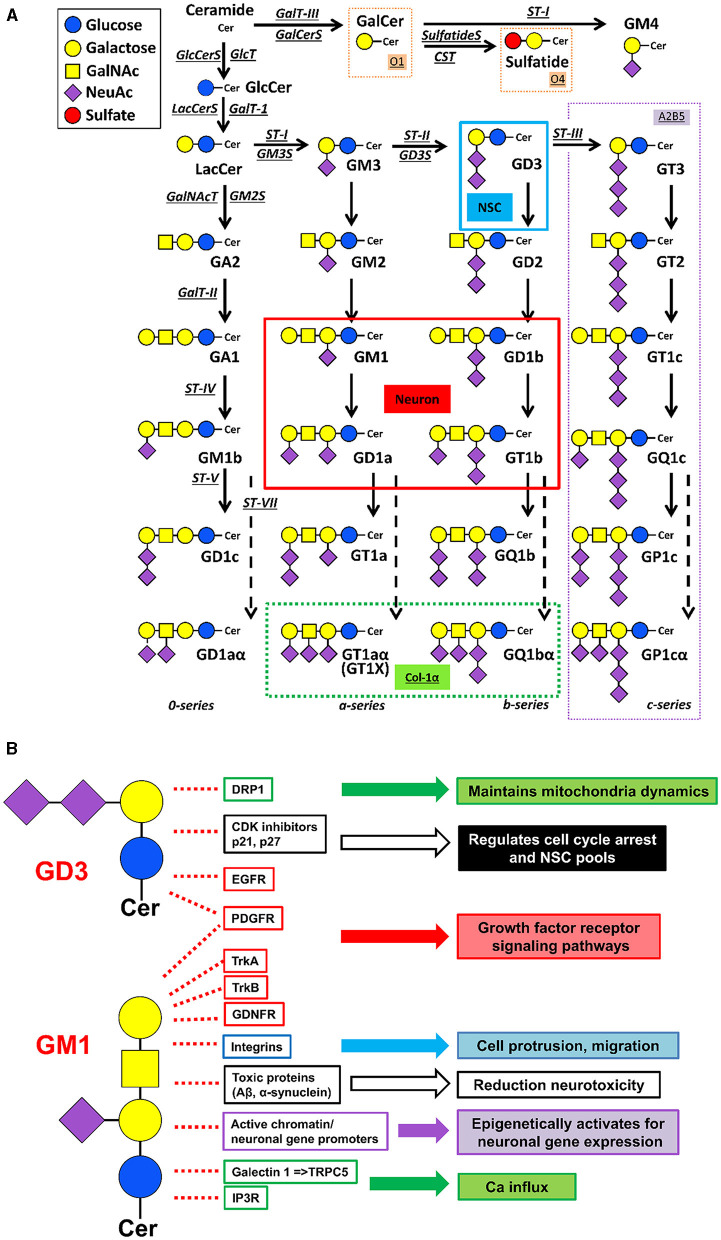
Metabolic pathways and functional roles of glycosphingolipids, including gangliosides. **(A)** Metabolic pathways and structure. Cer ceramide, CST cerebroside sulfotransferase (*Gal3st1*, sulfatide synthase), GalNAc-T N-acetylgalactosaminyltransferase I (*B4galnt1*, GA2/GM2/GD2/GT2 synthase), GalT-I galactosyltransferase I (*B4galT5 & B4galt6*, lactosylceramide synthase), GalT-II galactosyltransferase II (*B3galt4*, GA1/GM1/GD1b/GT1c synthase), GalT-III galactosyltransferase III (*Ugt8a*, galactosylceramide synthase), GlcT glucosyltransferase (*Ugcg*, glucosylceramide synthase), ST-I sialyltransferase I (*St3gal5*, GM3 synthase), ST-II sialyltransferase II (*St8Sia1*, GD3 synthase), ST-III sialyltransferase III (*St8Sia3*, GT3 synthase), ST-IV sialyltransferase IV (*St3gal2*, GM1b/GD1a/GT1b/GQ1c synthase), ST-V sialyltransferase V (*St8sia5*, GD1c/GT1a/GQ1b/GP1c synthase), ST-VII sialyltransferase VII (St6galnac6, GD1aα/GT1aα/GQ1bα/GP1cα-synthase). Official symbols of genes are represented in italics in this figure legend. GD3 is the most abundant ganglioside in neural stem cells (NSCs). c-Series gangliosides are A2B5 antigens. GM1, GD1a, GD1b, and GT1b are the most abundant ganglioside species in adult mammalian brain and neurons. Oligodendrocyte markers O1 and O4 are GalCer and sulfatide, respectively. GT1aα and GQ1bα are cholinergic-specific antigens (Chol-1α). **(B)** Examples of ganglioside functions. Without GD3, Drp1 levels are increased, and aberrant mitochondrial fragmentation is augmented. GD3 suppresses p21 expression and maintains p27 expression in NSCs. Gangliosides bind to the neurotrophic factor receptors to regulate their signalings. GM1 promotes integrin signaling for cell protrusion and migration. Gangliosides prevent and even reduce the accumulation of toxic proteins [e.g., amyloid-beta peptides (Aβs) and alpha-synuclein (α-syn)] in neurodegenerative diseases. Nuclear GM1 epigenetically promotes neuronal gene expression to sustain healthy neuronal functions. Elevation of GM1 levels induces Ca influx and neurite outgrowth. The listed in the figure is a limited examples, but, ganglioside-based multifunctional therapy is promising.

Patients with ganglioside-synthase mutations have symptomatic epilepsy syndrome with severe neuronal dysfunction or hereditary spastic paraplegias with additional neurological symptoms (reviewed, Itokazu et al., 2023). A majority of patients with neurodegenerative diseases have alterations in their glycosphingolipid metabolism and, those changes may contribute to their accompanying pathogenesis. Concentrations of complex gangliosides such as GM1 are significantly lower in the brains of patients with AD and PD (Wu et al., 2012; Hadaczek et al., 2015; Ariga, 2017; Seyfried et al., 2018). Some glycosphingolipid-synthase-knockouts (KO) are embryonic lethal. GM2 synthase (*GM2S*) is one of the key enzymes needed for synthesis of the major brain-type gangliosides, including GM1. GM2S-KO mice exhibit impaired movement and have virtually all the neuropathological symptoms of PD and cognitive impairment (Wu et al., 2011, 2012, 2020). GD3 synthase (GD3S)-KO mice led to depression-like behaviors and memory defect (Wang et al., 2014; Tang et al., 2021). With regard to the cholinergic system, Chol-1α gangliosides (GT1aα and GQ1bα, see Figure 1A) are minor species in the brain and serve as unique markers of cholinergic neurons (Ando et al., 1992; Hirabayashi et al., 1992; Itokazu and Yu, 2023). Chol-1α gangliosides may support cognitive functions such as memory and learning, and the administration of Chol-1α gangliosides appeared to alleviate the decreased synaptic functions in aged brains (Ando, 2014). GT1aα and GQ1ba, were found to be elevated in the brains of the patients with AD and AD model mice (Ariga et al., 2013; Ariga, 2017; Fukami et al., 2017). Neural stem cells (NSCs), non-differentiated precursor cells defined by their capacity for self-renewal and mulitpotency, express Chol-1α gangliosides (Ngamukote et al., 2007). Chol-1α antigens may play an important role in cholinergic synaptic transmission and participate in cognitive function, although the detailed mechanisms need to be determined. Overall, ganglioside deficiency leads to development of neurodegenerative diseases and mental health disorders, and a proper ganglioside composition may rescue the disease phenotypes.

Effects of organophosphates on glycolipid compositions have been reported. Subcutaneously injected organophosphate, diisopropylphosphorofluoridate (DFP) into chickens, resulted in an increase in GQ1b and GT1b levels but the proportion of GD3 decreased, while the concentrations of protein, total lipid, total cholesterol, and phospholipid were not affected (Bush et al., 1995). These progressive changes in ganglioside composition correlated with increasing ataxia. Intraperitoneal administration of the organophosphate, metasystox depleted levels of gangliosides in cerebral hemisphere, cerebellum, brain stem and spinal cord of rats (Islam et al., 1983). Other studies also reported that organophosphate effects on glycolipid expression in the CNS of mammals and birds (Rozengart and Taranova, 1969; Taranova, 1978; Khan and Hasan, 1988). As the organophosphate-induced alteration of ganglioside composition may impact brain dysfunction, the mechanisms of ganglioside changes after the administration of organophosphate need to be determined.

### 2.4 Curative gangliosides are expected to protect from CNS damages against chemical threat

An often overlooked but key aspect for successful translational studies is that functional activities of proteins and genes are highly dependent upon their molecular environments. Cells and their subcellular organelles are surrounded by biological lipid membranes that define their individual cellular shape and maintain cellular organization as well as provide functional platforms for cellular signaling (reviewed, Itokazu and Yu, 2023). Specific gangliosides control molecular functions on biological membranes, including the plasma, mitochondrial, and nuclear membranes. For example, GD3 is involved in the maintenance of NSC fate determination by interacting with epidermal growth factor receptors (EGFRs), by modulating expression of cyclin-dependent kinase (CDK) inhibitors p27 and p21, and by regulating mitochondrial dynamics via associating a mitochondrial fission protein, the dynamin-related protein-1 (Drp1) (Wang and Yu, 2013; Tang et al., 2021; Fuchigami et al., 2023, 2024). The postnatal NSC pools are declined in GD3S-KO mice, resulting in depressive symptoms, olfactory dysfunction, and impaired memory, with the deficiency of postnatal neurogenesis (Wang and Yu, 2013; Itokazu et al., 2018; Tang et al., 2021; Fuchigami et al., 2024). On the other hand, intranasal or intracerebroventricular infusion of GD3 restored postnatal NSC pools of GD3S-KO and AD mice (Itokazu et al., 2019; Fuchigami et al., 2023). A major ganglioside, GM1 promotes neuronal differentiation by an epigenetic regulatory mechanism (Tsai and Yu, 2014; Itokazu et al., 2016; Tsai et al., 2016). Nuclear GM1 binds with acetylated histones on the promoters of *GM2S*, a critical enzyme for GM1 synthesis, as well as on the *NeuroD1* genes in differentiating neurons. Further, intranasal GM1 infusion increased nuclear expression of nuclear receptor-related 1 (Nurr1), an essential transcription factor for differentiation, maturation, and maintenance of midbrain dopaminergic neurons in A53T and GM2S-KO mice (Itokazu et al., 2021). GM1 induces epigenetic activation of the tyrosine hydroxylase (*TH*) gene, including augmentation of acetylated histones and recruitment of Nurrr1 to the *TH* promoter region. To attenuate the effects of neurotoxic proteins in PD model mice, in our exciting data, nasal administration of gangliosides GM1 and/or GD3 reduced the neurotoxic α-synuclein levels and restored mitochondrial functions (Itokazu et al., 2021, 2023). Furthermore, exogenous GM1 seems beneficial to attenuate chronic microglia activation and neuroinflammation in neurodegenerative diseases (Galleguillos et al., 2022). GM1 also facilitates internalization of toxic proteins, such as α-synuclein into microglia through GM1-mediated endocytosis (Park et al., 2009). Pretreatment with intracerebroventricular infusion of GM1 for 4 days reduced organophosphate-, soman-induced seizure-related brain damage, while intraperitoneal injection of GM1 failed to provide protection (Ballough et al., 1998). So far, intracerebroventricular administration is the most reliable method to deliver gangliosides into the brain, we developed a more convenient non-invasive delivery procedure by intranasal infusion of gangliosides with success (Itokazu et al., 2021; Fuchigami et al., 2023). Intranasal administration can be a suitable route for efficient and non-invasive means for delivery of gangliosides to the brain, reducing systemic exposure and potential undesirable side effects.

## 3 Conclusion

The rapid rise in elderly populations worldwide is resulting in a dramatic increase in the incidence of age-related neurodegenerative illnesses. Currently, we lack both an understanding of the causes of these illnesses as well as how to effectively treat them. There is increasing interest in how environmental exposures over the lifespan might contribute to these diseases as well as the development of effective therapeutic interventions that are truly disease modifying and not just designed to provide temporary symptomatic relief. The purpose of this review was thus (1) to introduce one major environmental factor, toxicant (more specifically organophosphate) exposure and how it may lead to neurodegenerative illnesses, and (2) to introduce the role of gangliosides in the etiology of neurodegenerative diseases, how they may interact with organophosphates, and how exogenously administered gangliosides (especially by the intranasal route) might have diseases modifying capabilities for people who have neurodegenerative illnesses resulting from organophosphate exposures as well as other toxic insults. Gangliosides undergo dynamic qualitative and quantitative developmental, age-related, and pathological changes that correlate with neuronal function. Ganglioside microdomains provide a functional platform for cellular signaling of interacting molecules (Figure 1B). Progressive imbalance of cell membrane lipid composition is a physicochemical property that changes during normal aging, but further disruptions are observed in neurodegenerative diseases. A key aspect for successful translational studies is that the functional activities of proteins and genes are highly dependent upon their molecular environments, such as glycolipid micodomains. Multifunctional gangliosides can modulate protein and gene activities on plasma, mitochondrial, and nuclear membranes, in theory, may restore functions of specific molecules in the brains of patients with chemical-induced neurodegenerative diseases and mental health disorders.

## Author contributions

YI: Conceptualization, Funding acquisition, Investigation, Resources, Writing—original draft, Writing—review & editing. AT: Conceptualization, Investigation, Project administration, Resources, Supervision, Writing—review & editing.
